# *C. elegans* spermatocyte divisions show a weak spindle checkpoint response

**DOI:** 10.1242/jcs.257675

**Published:** 2024-03-27

**Authors:** Shang-yang Chen, Pu-wei Cheng, Hsiao-fang Peng, Jui-ching Wu

**Affiliations:** ^1^ Department of Clinical Laboratory Science and Medical Biotechnology, College of Medicine, National Taiwan University, Taipei 10048, Taiwan; ^2^ Department of Laboratory Medicine, National Taiwan University Hospital, Taipei 10048, Taiwan

**Keywords:** Spindle assembly checkpoint, Male meiotic divisions, Chromosome segregation, Securin, Proteasome

## Abstract

Male meiotic division exhibits two consecutive chromosome separation events without apparent pausing. Several studies have shown that spermatocyte divisions are not stringently regulated as in mitotic cells. In this study, we investigated the role of the canonical spindle assembly (SAC) pathway in *Caenorhabditis elegans* spermatogenesis. We found the intensity of chromosome-associated outer kinetochore protein BUB-1 and SAC effector MDF-1 oscillates between the two divisions. However, the SAC target securin is degraded during the first division and remains undetectable for the second division. Inhibition of proteasome-dependent protein degradation did not affect the progression of the second division but stopped the first division at metaphase. Perturbation of spindle integrity did not affect the duration of meiosis II, and only slightly lengthened meiosis I. Our results demonstrate that male meiosis II is independent of SAC regulation, and male meiosis I exhibits only weak checkpoint response.

## Teaser

**Highlighted Article:** *C. elegans* male meiosis II exhibits abridged spindle checkpoint pathway and its progression is independent of proteasome activities, whereas male meiosis I responds weakly to the spindle checkpoint.

## INTRODUCTION

**Fig. 1.**
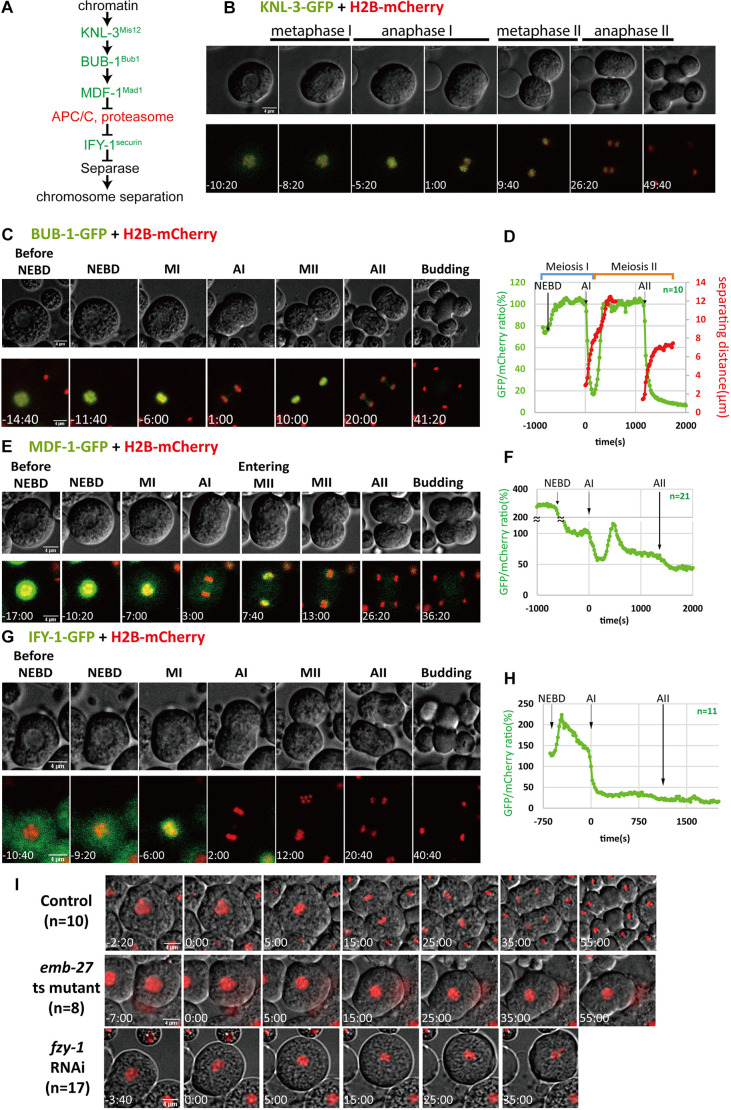
**Dynamics of chromatin-associated kinetochore and checkpoint proteins during male meiotic divisions.** (A) Components of the canonical SAC pathway tested in this study. Molecules labeled in green: dynamics to be examined using GFP transgenes. Molecules labeled in red: phenotypes of inhibition observed. Names given in superscript are those for the vertebrate homologs. (B,C,E,G) Representative time-lapse images of male meiotic division from spermatocytes expressing (B) KNL-3–GFP (*n*=10), (C) BUB-1–GFP (*n*=10), (E) MDF-1–GFP (*n*=21), and (G) IFY-1–GFP (*n*=11). mCherry–histone H2B were expressed in these strains for stage identification. Scale bars: 4 μm. (D) A representative graph showing the change of chromosome-associated BUB-1–GFP levels during male meiotic divisions. The trend of chromosome separation during two anaphase events are also shown. (F) Representative quantification of MDF-1–GFP intensity during male meiotic divisions. (H) Representative quantification of IFY-1–GFP intensity during male meiotic divisions. (I) Representative time-lapse images of control, *emb-27(ye143)* and *fzy-1(RNAi)* spermatocytes during male meiotic divisions. mCherry–histone H2B was used to label dividing chromosomes. MI, metaphase I; AI, anaphase I; MII, metaphase II; AII, anaphase II. Times are given as minutes:seconds relative to NEBD.

Faithful chromosome segregation during cell division is essential for normal development of multicellular organisms. The evolutionarily conserved spindle assembly checkpoint (SAC) pathway has been established as the key quality control system for the fidelity of cell division in mitotic cells (reviewed in Foley and Kapoor, 2013; Jia et al., 2013; Lara-Gonzalez et al., 2012; Musacchio and Salmon, 2007; Fig. 1A). During the early phase of cell division, the kinetochore acts as a platform to recruit SAC effectors Mad1 and Mad2 to the surface of chromosomes that have not been captured by the spindle microtubules. The kinetochore-bound SAC effectors form the MCC complex that sequesters Cdc20, an activator of the E3 ubiquitin ligase APC/C. The establishment of stable microtubule–chromosome interaction allows the release of SAC effectors and Cdc20. Cdc20 in turn activates APC/C and induces degradation of securin, the separase inhibitor, resulting in the activation of separase that cleaves chromosomal cohesion and therefore initiates chromosome separation. The SAC pathway ensures separation initiates only when chromosomes are properly captured by the spindle microtubules.

In contrast to mitosis, male meiosis exhibits two consecutive symmetric divisions to separate homologous and sister chromosomes sequentially, resulting in four haploid sperm. Although male meiotic divisions are often described as an iteration of mitotic division, it remains elusive whether the divisions are also subjected to stringent regulation. In most animals, the males harbor a pair of heterozygous sex chromosomes. During spermatogenesis in XY animals, as the autosomes are fully paired and synapsed, the sex chromosomes are only weakly synapsed through a pseudoautosomal region, due to a poor homology shared between X and Y chromosomes (reviewed in Kauppi et al., 2012). This causes a major problem for the canonical spindle assembly checkpoint program, because robust spindle microtubule attachments and tension establishment are prerequisites for checkpoint silencing. Therefore, it is intriguing how spermatocytes circumvent such a dilemma if the SAC pathway is present. In line with this, several studies suggested that male meiotic division is not stringently regulated as in mitotic cells. Individuals who suffer from human meiotic division deficiency-related male infertility generate macronuclear sperm containing tetraploid chromosomes and multiple flagella, indicating the male meiotic divisions can proceed with defective chromosome attachment (Dieterich et al., 2007; Escalier, 2002). Consistent with this, mice lacking one of the two copies of checkpoint genes BUB3 or MAD2 do not show elevated rates of aneuploid sperm, despite these mice showing significantly increased mis-segregation in somatic cells (Jeganathan and van Deursen, 2006). Furthermore, in fruit flies, misaligned or unpaired chromosomes only cause a delay in the initiation of chromosome separation in spermatocytes (Rebollo and Gonzalez, 2000).

In this study, we sought to evaluate the requirement of the canonical SAC pathway in both male meiotic divisions. The spermatogenic program from the nematode *Caenorhabditis elegans* has been shown to be regulated by evolutionarily conserved cell cycle and cell division factors (Chu et al., 2006; Shakes et al., 2009). Distinct from mammals, *C. elegans* spermatocytes can be isolated and cultured in the absence of a somatic gonad, allowing tracking of the progress of divisions with time-lapse microscopy (Peters et al., 2010; Wu et al., 2012). We show that the SAC pathway is present in primary but not secondary spermatocytes. Nonetheless, the progression of neither of the two divisions was significantly stalled by compromised spindle integrity. Our results support the idea that spermatocytes might have evolved to diminish the regulation from the spindle checkpoint.

## RESULTS AND DISCUSSION

### Levels of chromosomal outer kinetochore protein BUB-1 oscillate between two divisions

To investigate the roles of the SAC pathway in male meiotic divisions, we first examined the dynamics of SAC-related components during spermatocyte divisions (Fig. 1A). Given that spermatogenic chromatin does not de-condense between the two divisions (Shakes et al., 2009), we hypothesized that the kinetochore, which serves as the platform for both spindle–chromosome attachment and SAC effector recruitment, would persist on the condensed chromosomes throughout the divisions. We found the inner kinetochore protein KNL-3 appeared to be associated with chromosomes throughout the divisions (Fig. 1B; Movie 1). By contrast, the outer kinetochore protein BUB-1 showed dynamic chromosome association patterns (Fig. 1C; Movie 2). Consistent with previous studies in mitotic cells, the signals of BUB-1–GFP accumulated at metaphase chromosomes and disappeared upon anaphase onset during both divisions (Moyle et al., 2014; Vleugel et al., 2015). At the transition between the two divisions, BUB-1–GFP re-accumulated at the surface of chromosomes. Quantification of chromosome-associated GFP signals revealed that a steep drop of chromosomal BUB-1–GFP coincided with the initiation of chromosome separation in both division events (Fig. 1C,D). Chromosomal BUB-1–GFP levels were regained at the end of anaphase I and were stably maintained during metaphase II, after which they disassociated from chromosomes at anaphase II and remained undetectable (Fig. 1C,D). These findings suggest that at least part of the outer kinetochore undergoes disassembly and re-assembly between the two male meiotic divisions.

### Partial re-establishment of MDF-1 dynamics after first male meiotic division

Previous studies have demonstrated that Mad1 and Mad2, which are part of the SAC pathway, are recruited to chromosomes by BUB-1 (Moyle et al., 2014). We found that the signals of chromosome-associated MDF-1–GFP (MDF-1 is the worm homolog of Mad1) drastically decreased after nuclear envelope breakdown (NEBD) (Fig. 1E, Movie 3), consistent with the dissociation of Mad1 during chromosome capture and biorientation (Moyle et al., 2014). MDF-1–GFP was present throughout metaphase I but dropped at the onset of anaphase I. The signals of MDF-1–GFP reappeared at chromosomes after anaphase I, with the kinetics comparable to BUB-1–GFP, but at a much lower intensity (Fig. 1F). During meiosis II, MDF-1–GFP again decreased drastically and maintained at low levels during metaphase II, after which the signals dropped to background levels after anaphase II (Fig. 1E,F). These results suggest that SAC is reassembled in between two male meiotic divisions as observed with BUB-1–GFP.

### Securin is absent from the second male meiotic division

We next examined the dynamics of the SAC target IFY-1 (the worm homolog of securin), the target of the SAC pathway that undergoes APC/C and proteasome-dependent degradation upon checkpoint satisfaction. Initially, IFY-1–GFP was diffusely distributed in the cytoplasm, but it was rapidly recruited to chromosomes after nuclear envelope breakdown (NEBD) and remained on the surface of the chromosome through metaphase I (Fig. 1G; Movie 4). Similar to our observations for MDF-1–GFP, chromosomal-associated IFY-1–GFP gradually reduced during metaphase I and quickly dissipated from the chromosome at anaphase I onset (Fig. 1G,H). These results support that the SAC pathway is active during male meiosis I division. Consistent with this, inhibition of either the APC/C component EMB-27 or the APC/C activator FZY-1 (the worm homolog of Cdc20) led to the arrest of male meiosis I at metaphase (Fig. 1I). Interestingly, IFY-1–GFP levels did not recover after the first division, and the chromosomes separated with minimal securin levels in meiosis II (Fig. 1G,H; Movie 4). Taken together, we conclude that although the SAC pathway exists in the first male meiotic divisions, the target of SAC signaling is absent from the second division. Thus far, several studies in yeasts and female meiosis systems have shown that securin is present in meiosis II, including in mouse, pig and *Xenopus* oocytes (Fan et al., 2006; Huo et al., 2006; McGuinness et al., 2009; Nabti et al., 2008; Salah and Nasmyth, 2000). It is plausible that the lack of securin in meiosis II is a unique feature in male meiosis.

### Proteasome-dependent protein degradation is essential for the first but not second male meiotic division

**Fig. 2.**
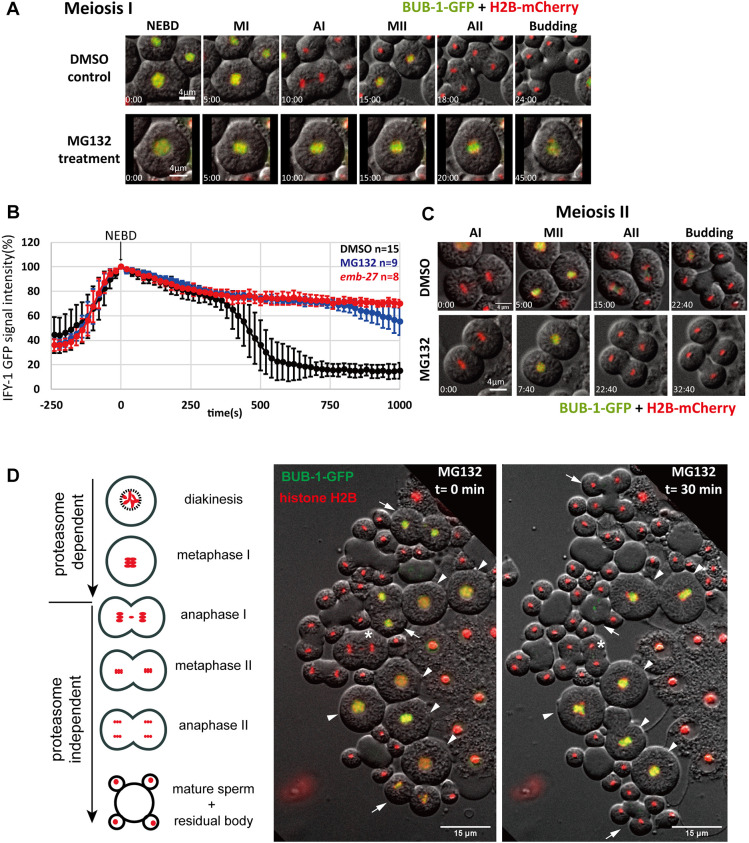
**Proteasome activities are required for first but not second male meiotic division.** (A) Representative time-lapse sequence of first male meiotic division with MG132 (*n*=19) versus DMSO (solvent) control (*n*=15). BUB-1–GFP is used for indication of metaphase nuclei. Scale bars: 4 μm. (B) Intensity profiles (mean±s.d.) of chromosomal associated IFY-1–GFP during first male meiotic division in wild type, *emb-27(ye143)* and MG132 treated primary spermatocytes. (C) Representative time-lapse images of second male meiotic division with MG132 (*n*=19) versus solvent control (*n*=10) in BUB-1–GFP-expressing worms. Scale bars: 4 μm. Times in A and C are given as minutes:seconds relative to NEBD. (D) A dissected male gonad with spermatocytes at various division stages (illustration and *t*=0 min) and after MG132 treatment for 30 min (*t*=30 min). Arrowheads, cells at start in at diakinesis or metaphase I; arrows, cells at start in metaphase II; asterisk, cell at start in anaphase I. Note absence of metaphase II spermatocytes at the end point for MG132 treatment. MI, metaphase I; AI, anaphase I; MII, metaphase II; AII, anaphase II.

The absence of securin in secondary spermatocytes suggests that degradation of securin is not the main mechanism that regulates male meiosis II division. To investigate this, we examined the roles of APC/C and proteasome activities in the second division. Previous studies have shown that temperature-sensitive APC/C mutations cause spermatocytes to arrest at metaphase I (Golden et al., 2000; Shakes et al., 2003); nonetheless, the brief transit between the two divisions meant that examination of second division challenging when using these mutants. To circumvent this, we took advantage of the heterogeneity of male worm germline. Dissected wild-type male gonad typically contains spermatogenic cells at different division stages (Shakes et al., 2009). We therefore could dissect male gonads into sperm medium containing proteasome inhibitor MG132 and track the division kinetics in spermatocytes that had undergone the first division. As expected, primary spermatocytes stopped at metaphase I, as evidenced by a lack of chromosome segregation and the persistence of BUB-1–GFP (Fig. 2A; Movie 5). Proteasome inhibition by MG132, as well as APC/C mutant *emb-27(ye143)*, caused the persistence of securin in primary spermatocytes, indicating failure to initiate chromosome separation (Fig. 2B). Surprisingly, when spermatocytes just past anaphase I were dissected into MG132-containing medium, BUB-1–GFP was able to reassemble on the chromosomes and the second division would normally take place (Fig. 2C; Movie 6). Furthermore, in dissected male gonads with cells at mixed-cell cycle stages, extended incubation with MG132 resulted in only stalled primary spermatocytes and mature sperm (Fig. 2D). These distinct responses between primary and secondary spermatocytes were not MG132 specific, as other proteasome inhibitors tested yielded the same results (Fig. S1). Our results demonstrate that proteasome activity is only required for the first and not the second male meiotic divisions.

### Progression of the second male meiotic division is insensitive to defective spindles

**Fig. 3.**
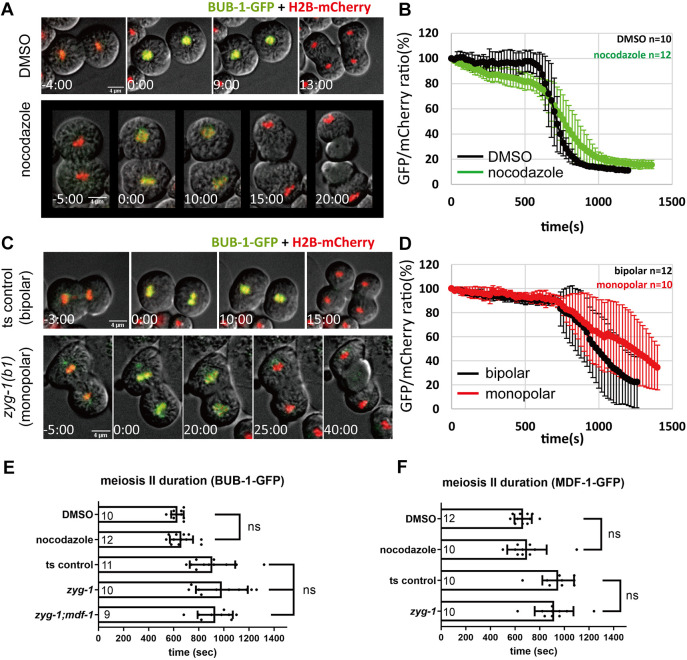
**Perturbation of spindle integrity does not affect dissociation timing of BUB-1 and MDF-1 in secondary spermatocytes.** (A) Progression of second division in BUB-1–GFP secondary spermatocytes treated with solvent control or nocodazole. (B) Kinetics of chromosome associated BUB-1–GFP in solvent or nocodazole-treated secondary spermatocytes. (C) Progression of second division in temperature shift control (bipolar) and *zyg-1(b1)* (monopolar) BUB-1–GFP secondary spermatocytes. γ tubulin–GFP is used to show cells with monopolar spindles, but not used for measurement of meiosis II duration. (D) Kinetics of chromosome-associated BUB-1-GFP in bipolar and monopolar secondary spermatocytes. Times for A–D are given as minutes:seconds or seconds relative to start of metaphase II. (E) Duration of meiosis II in control and spindle compromised secondary spermatocytes determined by persistence of BUB-1–GFP on chromosomes. (F) Duration of meiosis II in control and spindle compromised secondary spermatocytes determined by persistence of MDF-1–GFP. Results are mean±s.d. (*n* values as given in bars). ns, not significant (one-way ANOVA with an alpha of 0.05 with Bonferroni correction) Scale bars: 4 μm.

Because progression of the second male meiotic division is independent of proteasome activities, we were intrigued as to whether this would be affected when spindle integrity was perturbed. We used chromosomal BUB-1 residency as a readout for spindle checkpoint fulfillment, as has been shown in various mitotic systems (Bokros et al., 2016; Edwards et al., 2018; Logarinho and Bousbaa, 2008; Manic et al., 2017; Nerusheva et al., 2014). Nocodazole treatment depleted the bulk of spindle microtubules in both primary and secondary spermatocytes, resulting in the failure of chromosome segregation (Fig. S2). In nocodazole-treated secondary spermatocytes, chromosomes failed to separate and subsequently budded as diploid spermatids (Fig. S2). Despite the lack of spindles, we found that chromosome-associated BUB-1–GFP exhibited timely dissociation from non-aligned chromosomes in these cells (Fig. 3A). Quantification of chromosome-associated BUB-1–GFP revealed that, although BUB-1–GFP gradually departed from chromosomes, a timely sharp drop of BUB-1–GFP was still observed in spindleless secondary spermatocytes (Fig. 3B,E; Movie 6). Thus, the duration of metaphase II was not affected by the lack of spindle. In addition to depletion of spindle, we sought to examine the progression of the second male meiotic division with monopolar spindle, which yields half of kinetochores unattached and therefore should trigger spindle assembly checkpoint. Temperature-sensitive mutations in the Polo-like kinase ZYG-1 has been shown to cause centrosome duplication defects that result in monopolar spindles (O'Connell et al., 2001). We found that the temperature-sensitive *zyg-1(b1)* male worms exhibited an incomplete penetrance of phenotype, yielding both bipolar and monopolar primary and secondary spermatocytes (Fig. S3). BUB-1–GFP was present on chromosomes in monopolar secondary spermatocytes as in controls with bipolar spindles, which is consistent with the nocodazole experiments (Fig. 3C–E; Movie 7). We also examined the kinetics of MDF-1–GFP in secondary spermatocytes. In both spindle-depleted and monopolar spindle spermatocytes, MDF-1–GFP exhibited timely departure from chromosomes as in controls (Fig. 3F). Thus, perturbation of spindle integrity does not affect the progression of male meiosis II. Taken together, we conclude that male meiosis II does not have a checkpoint response.

### Perturbation of spindle integrity caused delayed male meiosis I

**Fig. 4.**
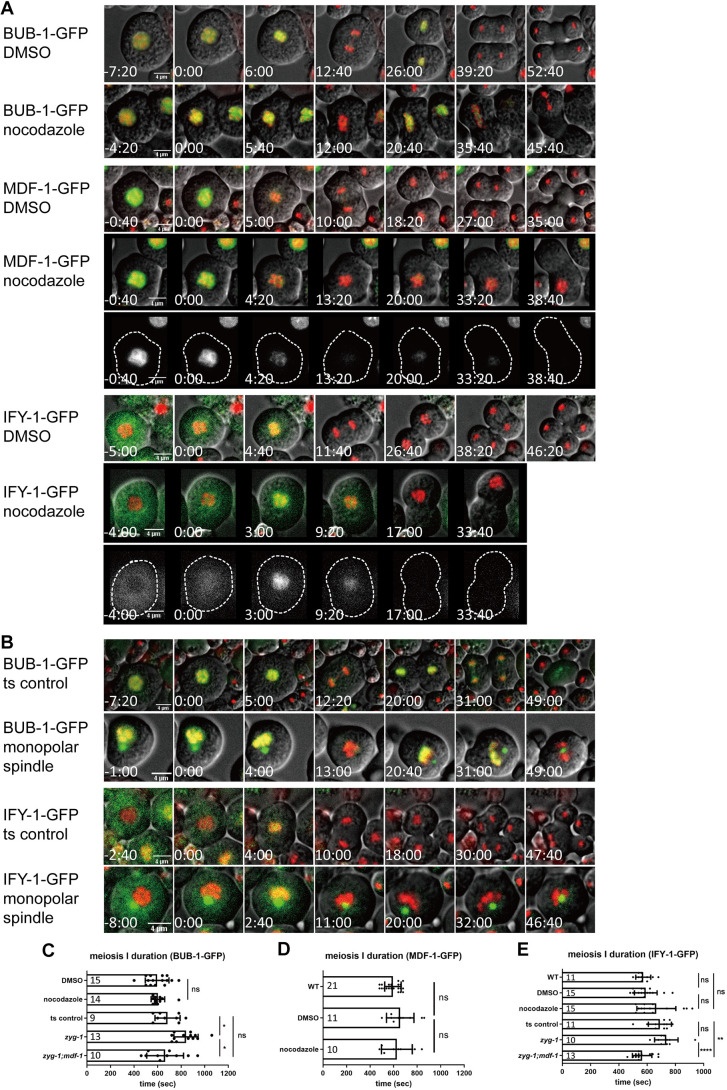
**Perturbation of spindle integrity did not cause metaphase I arrest in primary spermatocytes.** (A) Time-lapse images of cells expressing BUB-1–GFP (solvent control, *n*=15; nocodazole treatment, *n*=14), MDF-1–GFP (solvent control, *n*=11. nocodazole treatment, *n*=10) and IFY-1–GFP (solvent control, *n*=15; nocodazole treatment, *n*=15) during first male meiotic division. Dashed lines highlight the edge of cells. (B) Time-lapse images of cells expressing BUB-1–GFP (bipolar, *n*=11; monopolar spindle, *n*=10) and IFY-1–GFP (bipolar, *n*=11, monopolar spindle *n*=10) during first male meiotic division. γ tubulin-GFP is used to show cells with monopolar spindles, but not used for measurement of meiosis I duration. Times for A and B are given as minutes:seconds or seconds relative to NEBD. (C) Meiosis I duration in control and spindle-compromised primary spermatocytes determined by persistence of BUB-1–GFP on chromosomes from NEBD. (D) Chromosome association duration from NEBD in MDF-1–GFP-expressing primary spermatocytes with control or spindle perturbation. (E) Chromosome association duration from NEBD in IFY-1–GFP-expressing primary spermatocytes with control or spindle perturbation. Results are mean±s.d. (*n* values as given in bars). *P<0.005; **P<0.0005; ****P<0.0001; ns, not significant (one-way ANOVA with an alpha of 0.05). Scale bars: 4 μm.

Because components of the canonical SAC pathway are present in primary spermatocytes, and inhibition of proteasome caused a stall of division at metaphase I, we expected that perturbation of spindle integrity would induce a checkpoint response in primary spermatocytes. In nocodazole-treated primary spermatocytes, the chromosomes failed to align at the center of the primary spermatocytes (Fig. 4A,B; Movie 8). To our surprise, chromosomal BUB-1–GFP still exhibited oscillation patterns (Fig. 4A). The timing for dissociation of BUB-1–GFP in nocodazole-treated primary spermatocytes was comparable to that in the control (Fig. 4C). Furthermore, MDF-1–GFP and IFY-1–GFP in nocodazole-treated primary spermatocytes also exhibited kinetics that were indistinguishable from controls (Fig. 4A,D,E). Thus, despite the presence of the canonical SAC components, BUB-1, MDF-1 and IFY-1 can still be removed from chromosomes during the first male meiotic division when the microtubule-based spindle fails to form.

To exclude the possibility that the residual microtubules after nocodazole treatment were enough for interacting with chromosomes and triggering SAC silencing, we examined the dynamics of the SAC components in monopolar primary spermatocytes. In monopolar *zyg-1(b1)* spermatocytes, BUB-1–GFP also exhibited oscillation kinetics (Fig. 4B; Movie 9), suggesting that the cells were not arrested. However, BUB-1–GFP signals persisted on chromosomes significantly longer than in temperature-shifted controls, indicating the duration of meiosis I is prolonged (Fig. 4C). Such lengthening of meiosis I is dependent on SAC, for the chromosome residency of BUB-1–GFP in *zyg-1;mdf-1* mutant spermatocytes were comparable to the temperature-shifted controls (Fig. 4C). These results show that monopolar spindle indeed triggered checkpoint response during meiosis I. We were unable to examine the dynamics of MDF-1–GFP in this experiment since temperature shifts caused severe sickness in this transgenic strain. Although temperature shifting also slightly caused prolonged meiosis I in IFY-1–GFP transgenic spermatocytes, we could also observed a slight increase of IFY-1–GFP residence time in monopolar primary spermatocytes that can be reverted in *zyg-1;mdf-1* mutants (Fig. 4B,E). Taken together, we conclude that perturbation of spindle integrity triggers a weak checkpoint response in male meiosis I.

Our results support previous studies that spermatocytes do not exhibit a strong checkpoint response (Dieterich et al., 2007; Escalier, 2002; Jeganathan and van Deursen, 2006; Rebollo and Gonzalez, 2000).

## MATERIALS AND METHODS

### Strains

Worm strains used in this study are listed in Table S1. Worms were usually maintained at 20°C, except for temperature-sensitive strains (containing *ye143* or *b1* alleles), which were maintained at 15°C. Worms containing allele *emb-27(ye143)* were shifted to 25°C for 24 h from L4 larvae stage for experiments. Worms containing allele *zyg-1(b1)* were shifted to 26°C for 48 h from L2 larvae stage for experiments.

### RNA interference

The *fzy-1* RNAi sequence was provided by the *C. elegans* RNAi Library (Kamath and Ahringer, 2003). The primers for the *fzy-1* RNAi sequence were modified by adding a T7 promoter sequence on both the forward and reverse primer. The template was first produced by PCR from the worm genome, followed by TA cloning to insert the *fzy-1* RNAi sequence into the T&A™ Cloning Vector; finally, this plasmid was used for PCR and to get linearized DNA template for *in vitro* transcription. *In vitro* transcription was performed by using the MEGAscript^®^ kit (Thermo Fisher Scientific). The recovery of RNA was undertaken by lithium chloride precipitation as provided in the kit. dsRNA was injected directly into the gonad of strain XC38 male worms, then let the worms recover for 24 h. After 24 h, the worms were dissected and followed the time-lapse recording protocol to film the spermatocytes.

### Time-lapse recording

Male worms were dissected with a 22G needle on microscope in with a drop of complete sperm medium (50 mM HEPES pH 7.0, 45 mM NaCl, 25 mM KCl, 1 mM MgSO_4_, 5 mM CaCl_2_ and 1 mg/ml BSA). An 18×18 mm coverslip was then placed on the sample to squeeze the gonads for releasing spermatocytes. The edges of the coverslip were sealed with Vaseline to prevent evaporation of the medium. For small-molecule drug treatments, worms were dissected into complete sperm medium containing inhibitors with specific concentrations. The following inhibitors were used: nocodazole (Sigma) 1 μM, MG132 (Sigma) 10 μM, Bortezomib (Selleckchem) 10 μM, Carfilzomib (Selleckchem) 25 μM. Time-lapse images were captured under an Olympus IX83 microscope with an 100× objective and DIC optics every 20 s. The dynamics of chromosomes were followed in strains containing mCherry-*his-58* transgene. ImageJ (Fiji) was used for measuring the distance of the separate chromosomes, defined as the length from the endpoint of two chromosome masses. Anaphase onset was defined as the initial timing of visible separated chromosomes, both in anaphase I and II. NEBD was defined as the loss of round shape of the nucleus under DIC optics.

### Image processing and statistical analysis

Fluorescence intensity analyses were performed in ImageJ (Fiji). In brief, a mask was made from thresholded mCherry–histone H2B channel, which served as a region of interest for intensity measurement on the GFP channel. The statistical data was calculated by one-way ANOVA with an alpha of 0.05 and Bonferroni correction with Microsoft Excel 2013. Results were considered statistically different at *P*<0.05. Graphs were generated with Microsoft Excel or GraphPad Prism.

## Supplementary Material

10.1242/joces.257675_sup1 Supplementary information
