# Supplementary material for: C. elegans spermatocyte divisions show a weak spindle checkpoint response
Source: J Cell Sci. 2024 Mar 27;137(6):jcs257675. doi: 10.1242/jcs.257675 (PMC11651637; doi:10.1242/jcs.257675)
Supplement: Supplementary information [file joces-137-257675-s1.pdf]

Supplementary Figure 1

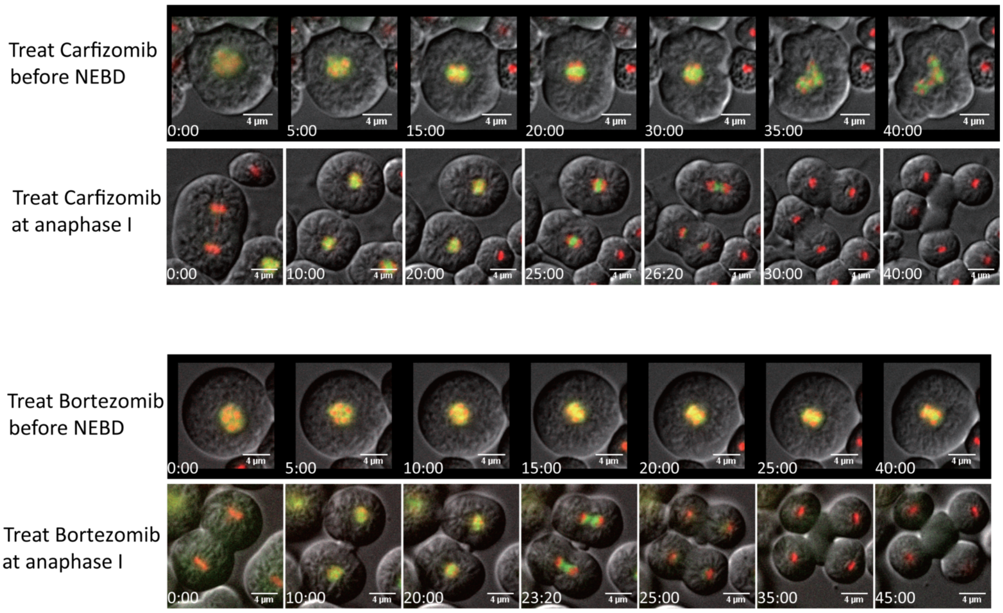

Fig. S1. Proteasome inhibitors caused arrest in meiosis I but not meiosis II in spermatocytes. Two additional proteasome inhibitors, Carfizomib and Bortezomib, were used to examine the progressio n of male meiotic divisions. scale bars: 4μm.

Journal of Cell Science • Supplementary information

Supplementary Figure 2

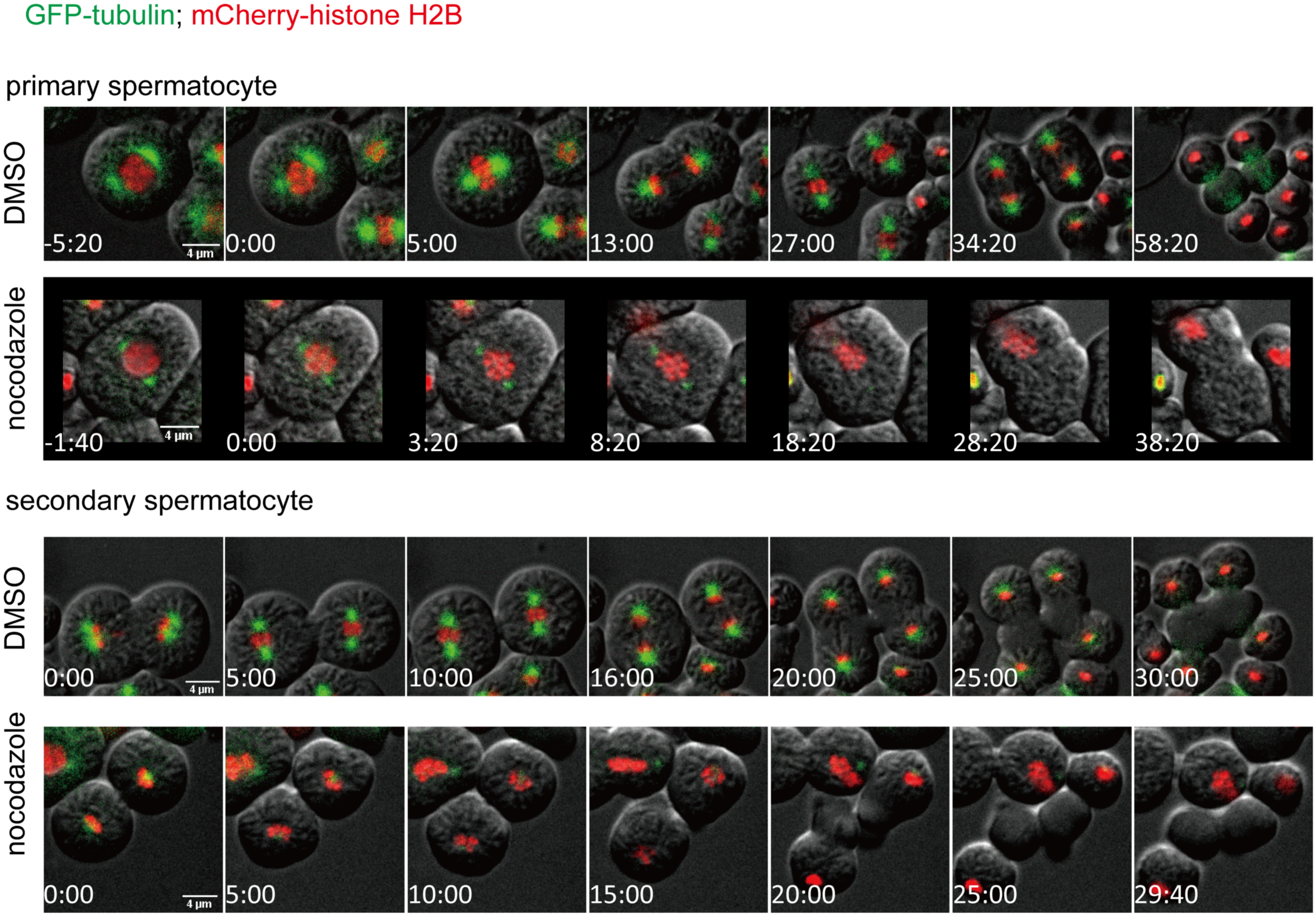

Fig. S2. Nocodazole induced depletion of the bulk of microtubule spindles in both primary and sec ondary spermatocytes.

Journal of Cell Science • Supplementary information

Supplementary Figure 3

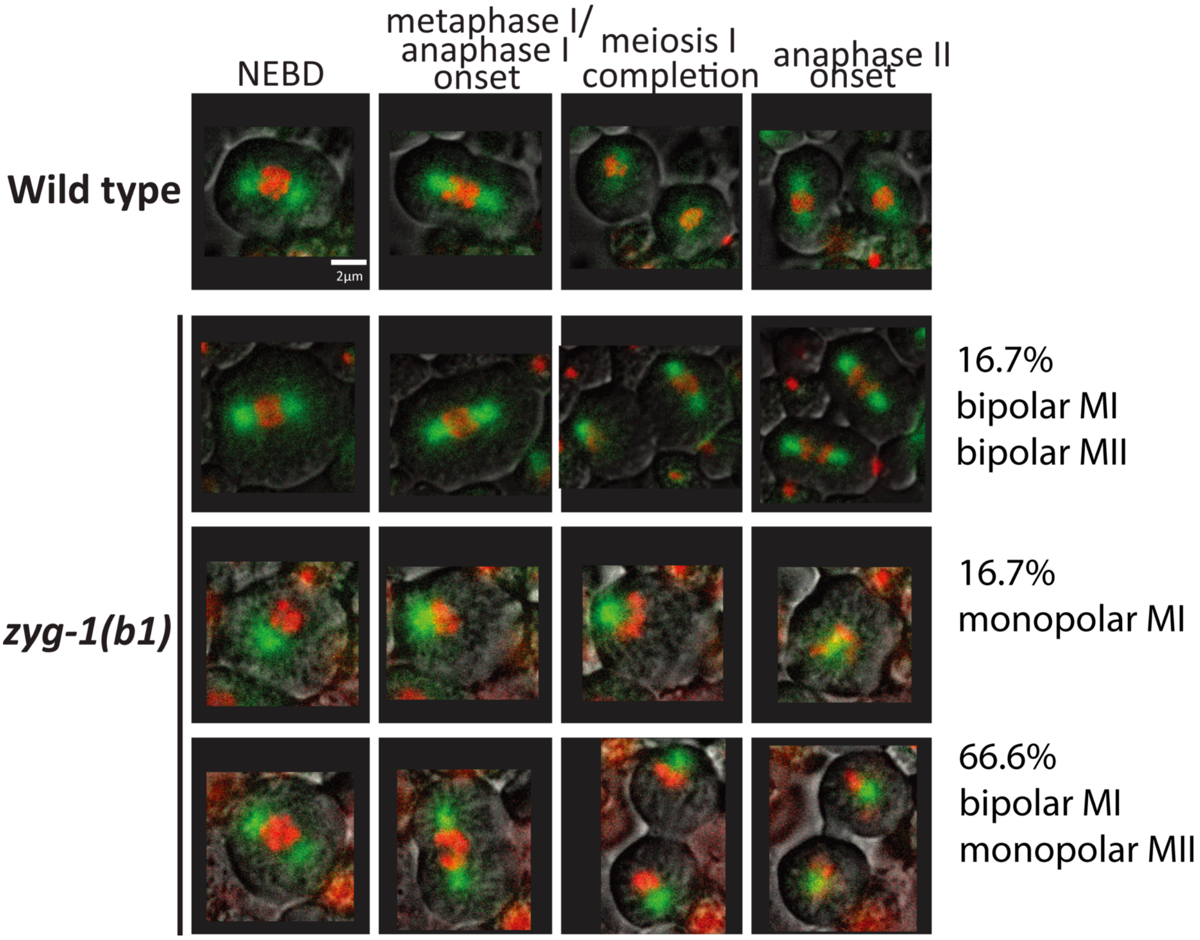

Fig. S3. *zyg-1(b1)* mutant exhibited incomplete penetrance of centrosome duplication defects. Table S1. Strained used in this study.

Table S1. Worm strains used in this study

**JCW17**	*ddIs68 [bub-1::TY1::EGFP::3xFLAG(92C12) + unc-119(+)]; ltIs37 [(pAA64) pie-1p::mCherry::his-58 + unc-119(+)] him-8 (e1489) IV*	Generated in this study
**JCW4**	*ruIs57[unc-119(+) pie-1::GFP::tubulin]; Itls37[pAA64; pie-1/mCherry::his-58; unc-119 (+)] him-8(e1489)IV*	Generated in this study
**JCW40**	*ltIs1[pIC22; pie-1 promoter::knl-3::GFP + unc-119(+)]; ltIs37 [(pAA64) pie-1p::mCherry::his-58 + unc-119(+)] him-8 (e1489) IV*	Generated in this study
**JCW45**	*ddIs128 [ify-1::TY1::EGFP::3xFLAG(92C12) + unc-119(+)] III; mCherry-his-58 him-8 IV*	Generated in this study
**JCW71**	*ddIs68 [bub-1::TY1::EGFP::3xFLAG(92C12) + unc-119(+)]; tpeSi3[Ppie-1::mCherry-his-10 cbr-unc-119(+)] II; him-5 V*	Generated in this study
**JCW103**	*zyg-1(b1)II; ddIs68 [bub-1::TY1::EGFP::3xFLAG(92C12) + unc-119(+)]; ltIs37 [(pAA64) pie-1p::mCherry::his-58 + unc-119(+)] him-8 (e1489) IV; ddIs6 [pie-1p::GFP::tbg-1 + unc-119(+)] V.*	Generated in this study
**JCW106**	*ddIs68 [bub-1::TY1::EGFP::3xFLAG(92C12) + unc-(+)]; ltIs37 [(pAA64) pie-1p::mCherry::his-58 + unc-119(+)] him-8 (e1489) IV; ddIs6 [pie-1p::GFP::tbg-1 + unc-119(+)] V.*	Generated in this study
**JCW113**	*zyg-1(b1)II; ddIs128 [ify-1::TY1::EGFP::3xFLAG(92C12) + unc-119(+)] III; ltIs37 [(pAA64) pie-1p::mCherry::his-58 + unc-119(+)] him-8 (e1489) IV; ddIs6 [pie-1p::GFP::tbg-1 + unc-119(+)] V.*	Generated in this study
**JCW112**	*zyg-1(b1)II; ddIs68 [bub-1::TY1::EGFP::3xFLAG(92C12) + unc-119(+)]; ltIs37 [(pAA64) pie-1p::mCherry::his-58 + unc-119(+)] him-8 (e1489) IV; unc-46(e177) mdf-1(gk2) V/nT1 [qIs51] (IV;V)*	Generated in this study
**JCW121**	*him-8(e1489) Itls37[pAA64; pie-1/mCherry::his-58; unc-119 (+)] IV; mad-1(lt39[gfp::tev::loxP::3xFlag::mad-1])V*	Generated in this study
**JCW122**	*zyg-1(b1)II; ddIs128 [ify-1::TY1::EGFP::3xFLAG(92C12) + unc-119(+)] III; ltIs37 [(pAA64) pie-1p::mCherry::his-58 + unc-119(+)] him-8 (e1489) IV*	Generated in this study
**JCW130**	*zyg-1(b1)II; him-8(e1489) Itls37[pAA64; pie-1/mCherry::his-58; unc-119 (+)] IV; mad-1(lt39[gfp::tev::loxP::3xFlag::mad-1])V*	Generated in this study

**JCW131**	*zyg-1(b1)II; ddIs68 [bub-1::TY1::EGFP::3xFLAG(92C12) + unc-119(+)]; ltIs37 [(pAA64) pie-1p::mCherry::his-58 + unc-119(+)] him-8 (e1489) IV*	Generated in this study
**JCW148**	*zyg-1(b1)II; ddIs128 [ify-1::TY1::EGFP::3xFLAG(92C12) + unc-119(+)] III; ltIs37 [(pAA64) pie-1p::mCherry::his-58 + unc-119(+)] him-8 (e1489) IV; unc-46(e177) mdf-1(gk2) V/nT1 [qIs51] (IV;V)*	Generated in this study
**JCW61**	*ddIs128 [ify-1::TY1::EGFP::3xFLAG(92C12) + unc-119(+)] III; emb-27(ye143) II;him-8(e1489) Itls37[pAA64; pie-1/mCherry::his-58; unc-119 (+)] IV*	Generated in this study
**JCW62**	*ddIs68 [bub-1::TY1::EGFP::3xFLAG(92C12) + unc-119(+)]; emb-27(ye143) II;him-8(e1489) Itls37[pAA64; pie-1/mCherry::his-58; unc-119 (+)] IV*	Generated in this study
**TH229**	*unc-119(ed3) III; ddIs68 [bub-1::TY1::EGFP::3xFLAG(92C12) + unc-119(+)]*	CGC
**TH214**	*unc-119(ed3) III; ddIs128 [ify-1::TY1::EGFP::3xFLAG(92C12) + unc-119(+)] III*	CGC
**OD1**	*unc-119(ed3) III; Itls1[pIC22; pie-1 promoter::knl-3::GFP + unc-119(+)]*	CGC
**KR3627**	*unc-46(e177) mdf-1(gk2) V/nT1 [let-?(m435)] (IV;V)*	CGC
**OD2906**	*mad-1(lt39[gfp::tev::loxP::3xFlag::mad-1])V*	The Desai Lab
**DH1**	*zyg-1(b1) II*	CGC
**HY621**	*emb-27(ye143) II*	CGC
**XC38**	*him-8(e1489) Itls37[pAA64; Ppie-1::mCherry::his-58; unc-119(+)] him-8(e1489) IV*	Chu's Lab (Wu et al., 2012)

Journal of Cell Science • Supplementary information

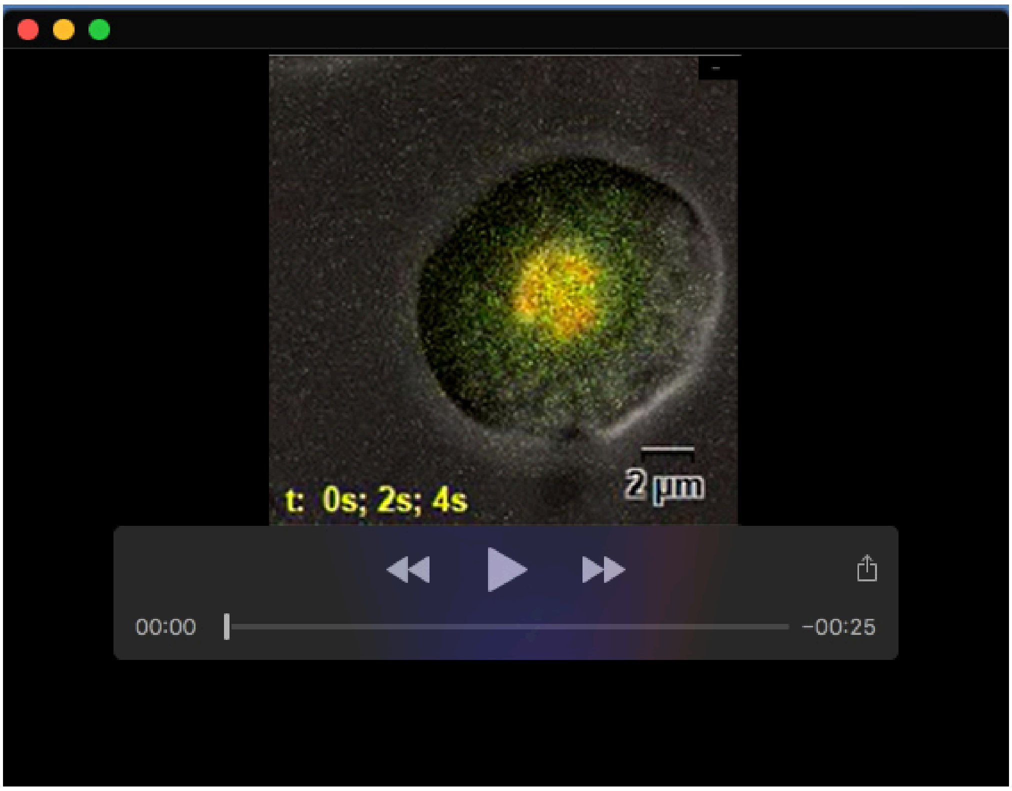

Movie 1. Male meiotic divisions in *C. elegans* spermatocyte expressing KNL-3-GFP and mCherryhistone H2B.

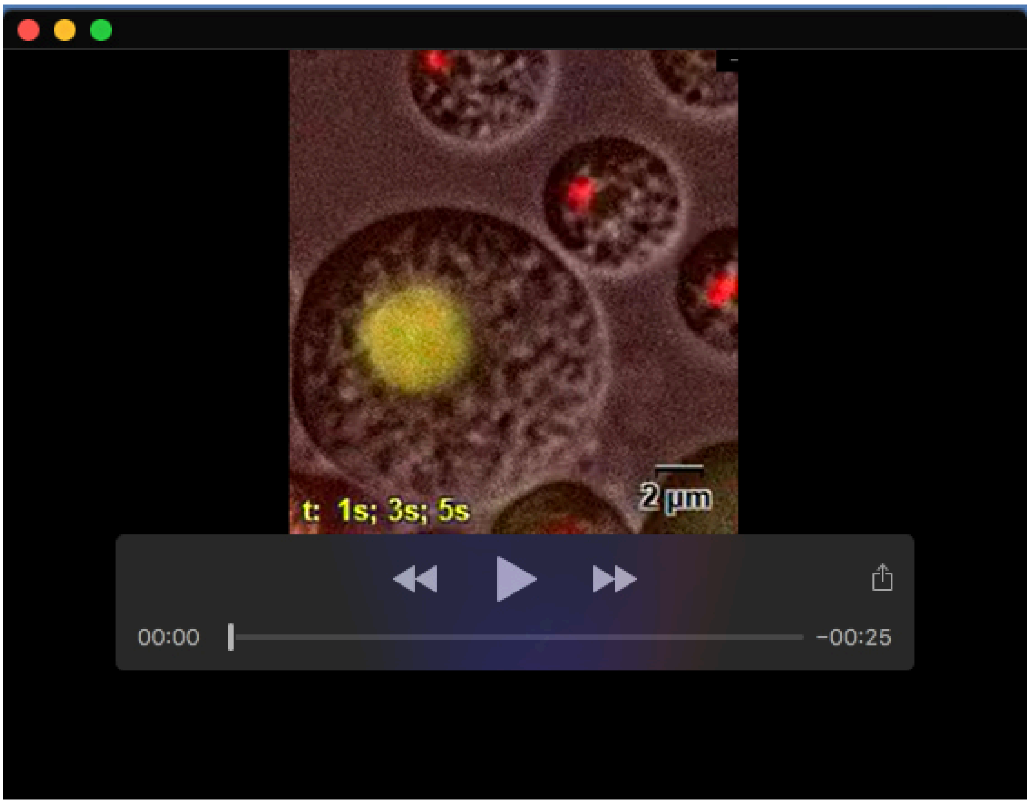

Movie 2. Male meiotic divisions in *C. elegans* spermatocyte expressing BUB-1-GFP and mCherryhistone H2B.

Journal of Cell Science • Supplementary information

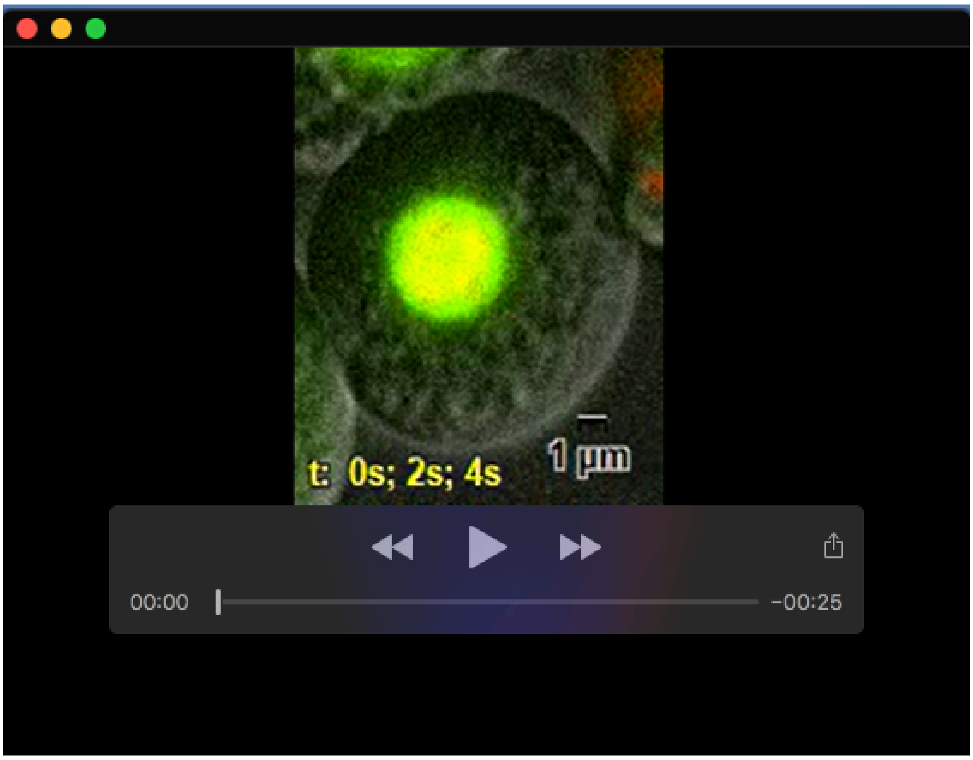

Movie 3. Male meiotic divisions in *C. elegans* spermatocyte expressing MDF-1-GFP and mCherry -histone H2B.

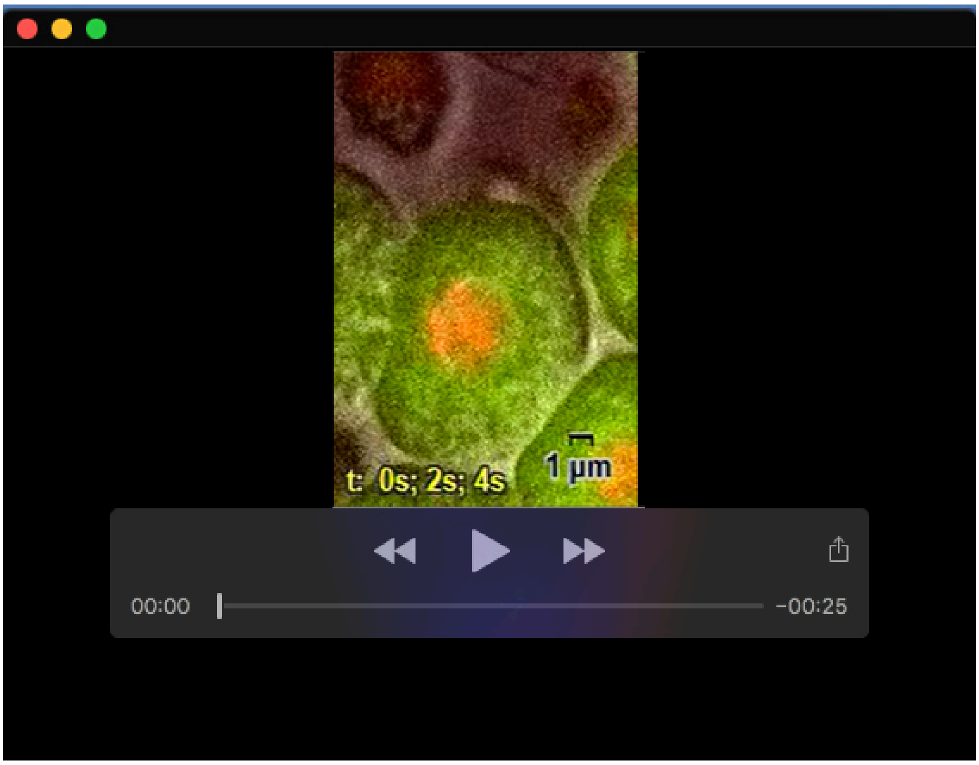

Movie 4. Male meiotic divisions in *C. elegans* spermatocyte expressing IFY-1-GFP and mCherryhistone H2B.

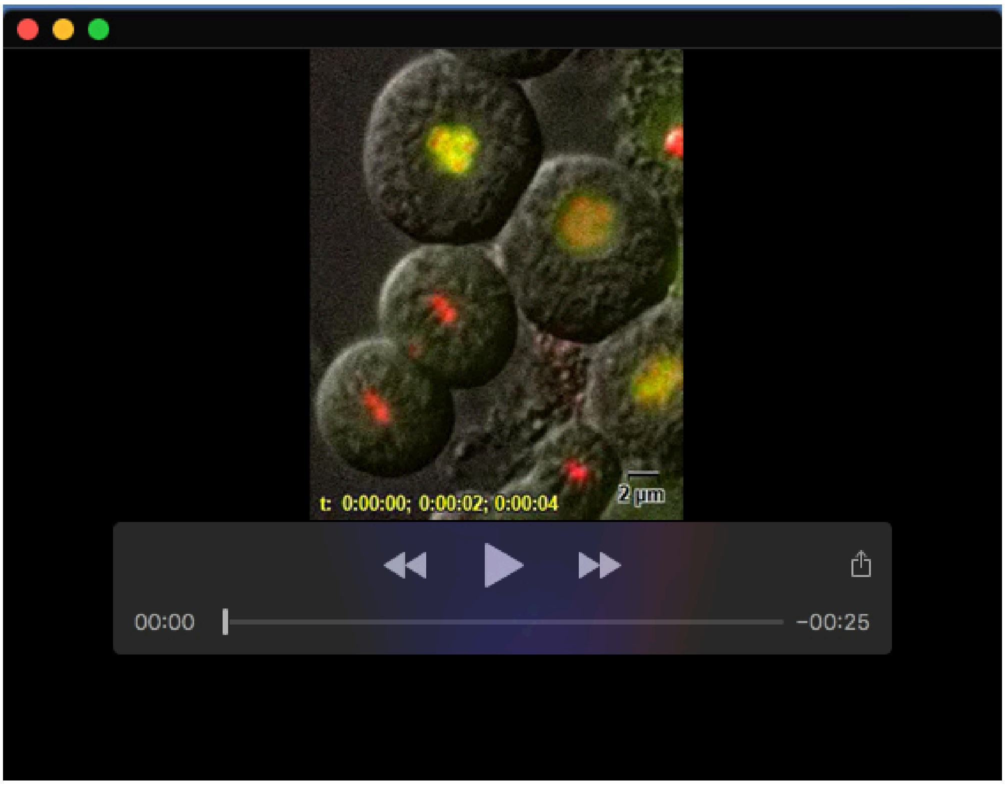

Movie 5. BUB-1-GFP and mCherry-histone H2B-expressing primary and secondary spermatocytes treated with MG132.

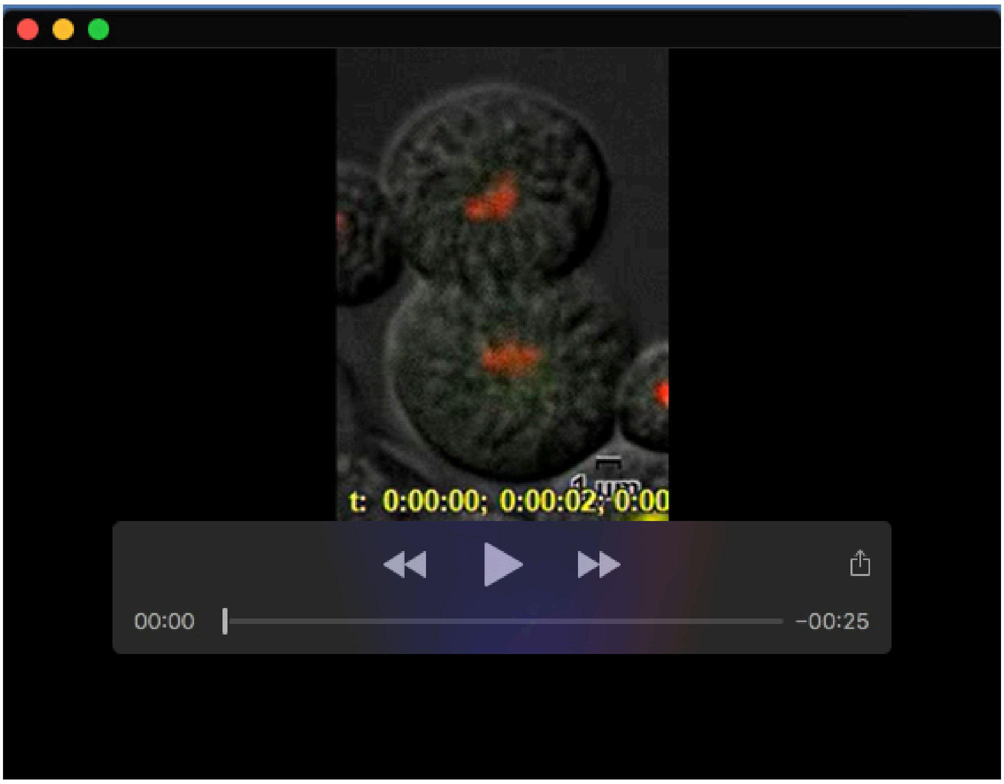

Movie 6. BUB-1-GFP and mCherry-histone H2B-expressing secondary spermatocytes treated with nocodazole.

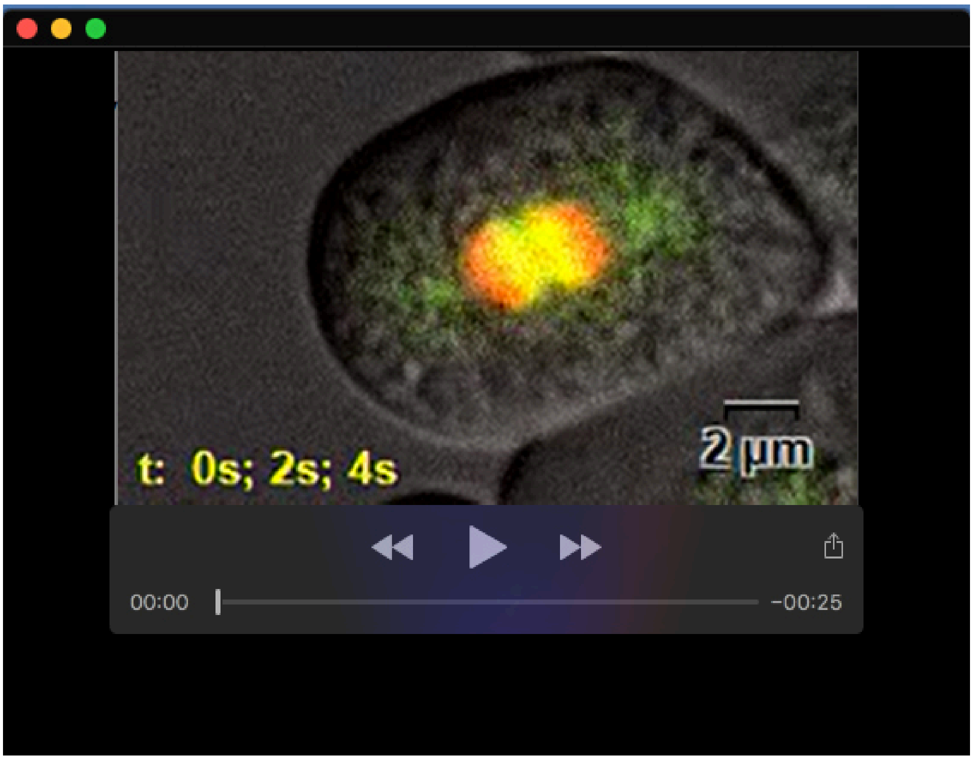

Movie 7. BUB-1-GFP, TBG-1-GFP and mCherry-histone H2B-expressing secondary spermatocyte with monopolar spindle.

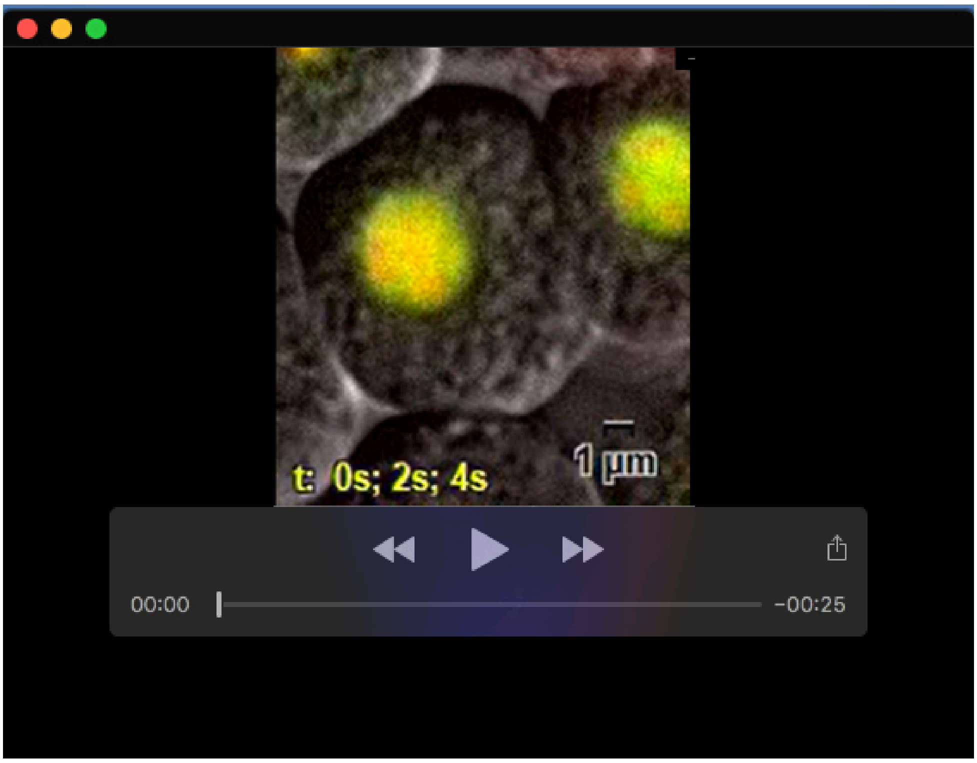

Movie 8. BUB-1-GFP and mCherry-histone H2B-expressing primary spermatocyte treated with nocodazole.

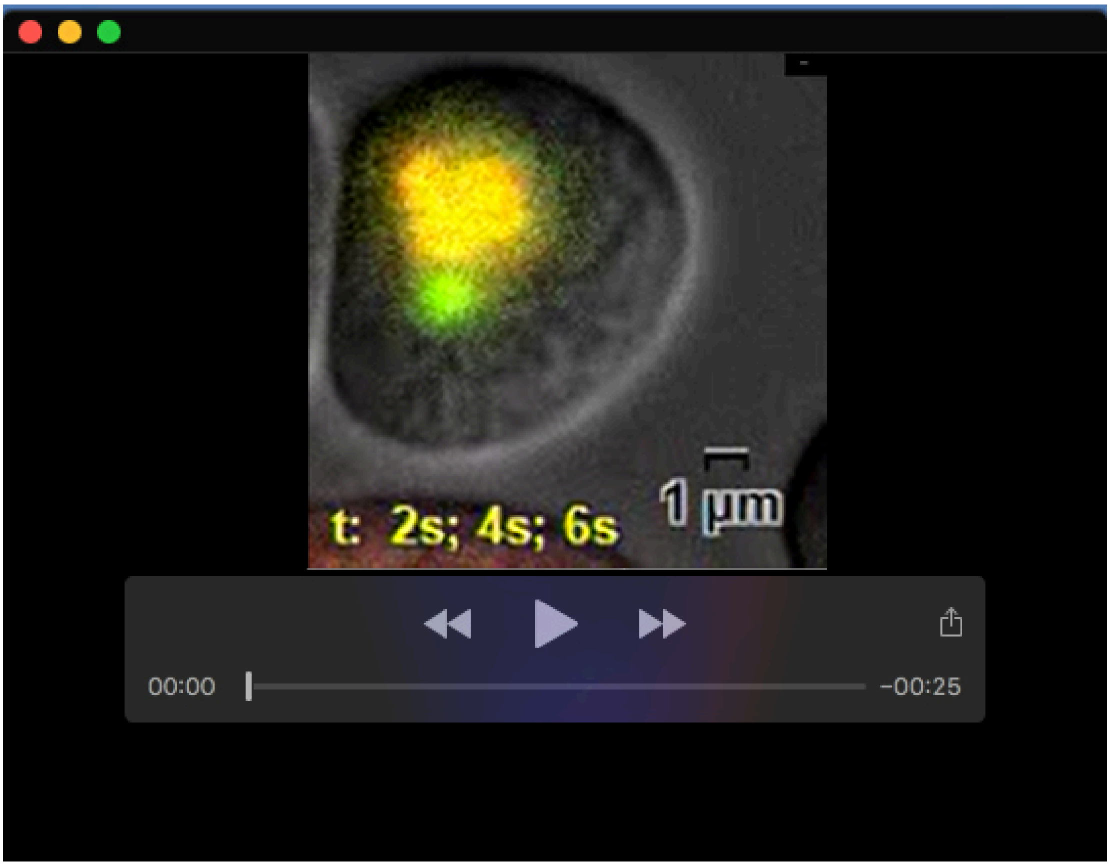

Movie 9. BUB-1-GFP, TBG-1-GFP and mCherry-histone H2B-expressing primary spermatocyte with monopolar spindle.
